# Toxicological Memoranda

**Published:** 1894-12-01

**Authors:** 


### TOXICOLOGICAL MEMORANDA.

[We shall be glad to receive, at our Office, 428, Strand, London, W.C., from the manufacturers, specimens of all new preparations and drugs which may be brought out from time to time.]

### HYPODERMIC USE OF MAGNESIUM SULPHATE.

Dr. J. H. Wade has used subcutaneous injection of magnesium sulphate in 46 cases. A 2 per cent. solution of the salt was employed, and quantities of it representing from about 2 to 4½ grains of magnesium sulphate were given. Two small doses at short intervals were more active than a single large dose. The injections were made under the skin of the arm, and never produced general or local disturbance. Out of 100 injections, 67 purgations occurred; one evacuation of the bowels took place 53 times, and two evacuations 10 times. The shortest time between administration and purgation was 3 hours, the longest 14 hours. This method of giving saline cathartics would probably prove of value in gastritis, unconsciousness, or where one did not wish to irritate the mucous membrane of the bowel.

### TRIKRESOL.

Professor Chartens[1] has published a short research on the action of trikresol. Crude carbolic acid consists chiefly of cresols, which are only slightly soluble in water owing to a large admixture of hydrocarbons, pyridene bases, and other similar bodies. If these be removed a clear liquid of pleasant odour is left, known as "trikresol," and which is a mixture of ortho-, meta-, and para-cresol. It is soluble in water to the extent of about 2½ per cent., and boils down between 185 and 205 deg. C. (365 to 401 deg. F.). In guinea pigs the lethal dose is about eight minims, and it causes convulsions similar to those produced by paraphenol, which it very closely resembles in action. Its toxic effect on micro-organisms was found to be about three times as strong as that of carbolic acid, while its general poisonous effect was only about one-third, which gives it a pronounced advantage over the latter as an antiseptic for surgical use. A ½ to 1 per cent. solution is strong enough for most purposes.

Professor Liebrich, of Berlin,[2] also speaks highly of its value as an antiseptic, and draws attention to the fact that lysol, solutol, solveol, and creolin owe their disinfecting properties to the cresols, which they contain in varying proportions.

[1] Lancet, 1894, p. 801. [2] Therap. Monastch., 1894, 25.

### ARSENIC IN GLYCERINE.

Attention has frequently been called to the probable presence of arsenic in glycerine, owing to contamination from the materials employed in its manufacture, and recently the public analyst of Leeds has stated that appreciable quantities are found in the glycerine of commerce. Paul and Cownley (Pharmac. Journal, Feb. 24th, 1894) have examined eight samples of glycerine taken at random with results of a very reassuring character. In three no arsenic was found; in two only one grain of metallic arsenic in fourteen pounds of glycerine, and in other two samples only one-tenth of this amount. In one sample which, however, was of a quality only used in making nitro-glycerine the amount was considerable. Marsh's test is not sufficiently delicate to detect such small quantities, and the authors proceeded as follows: 2 c.c. of glycerine were put in a long test tube with 5 c.c. of dilute hydrochloric acid and one gramme of pure zinc, while over the mouth of the test tube was placed a slip of filter paper moistened with mercuric chloride and then dried. If arsenic be present in any appreciable amount a yellow stain is produced on the paper in a few minutes, and it subsequently becomes darker. If no stain is produced in fifteen minutes the glycerine may be considered as arsenic-free. In two of the samples tested the arsenic only amounted to 1 part in 100,000, and in other two to 1 in 1,000,000. It is necessary that the reagents used should be quite pure, free from arsenic and also sulphur, as in the latter case sulphuretted hydrogen may be formed and give erroneous results.

